# Effect of Segment-Body Vibration on Strength Parameters

**DOI:** 10.1186/s40798-015-0022-z

**Published:** 2015-07-03

**Authors:** Ruben Tobias Goebel, Heinz Kleinöder, Zengyuan Yue, Ranajay Gosh, Joachim Mester

**Affiliations:** 1 Sport Science Program, Qatar University, P.O. Box 2713, Doha, Qatar; 2 Institute of Training Science and Sport Informatics, German Sport University Cologne, Cologne, Germany; 3 The German Research Center, Center of Elite Sport, German Sport University Cologne, Cologne, Germany; 4 Department of Mechanical and Industrial Engineering, Northeastern University, Boston, MA USA

**Keywords:** Segment-body vibration, Strength training, Maximum isometric force, Maximum range of motion, Maximum muscle tension

## Abstract

**Background:**

In this study, we examine the biomechanical advantage of combining localized vibrations to hamstring muscles involved in a traditional resistance training routine.

**Methods:**

Thirty-six male and female participants with at least 2 years of experience in resistance training were recruited from the German Sport University Cologne. The participants were randomized into two training groups: vibration training group (VG) and traditional training group (TTG). Both groups underwent a 4-week training phase, where each participant worked out at 70 % of the individual 1 repeat maximum (RM—maximum load capacity of a muscle for one lift to fatigue) (4 sets with 12 repetitions each). For participants in the VG group, local vibration was additionally applied directly to hamstring muscles during exercise. A 2-week examination phase preceded the pretests. After the pretests, the subjects underwent a prescribed training for 4 weeks. At the conclusion of the training, a 2-week detraining was imposed and then the study concluded with posttests and retest.

**Results:**

The measured parameters were maximum isometric force of the hamstrings and maximum range of motion and muscle tension at maximum knee angle. The study revealed a significant increase in maximum isometric force in both training groups (VG = 21 %, TTG = 14 %). However, VG groups showed an increase in their range of motion by approximately 2 %. Moreover, the muscle tension at maximum knee angle increased less in VG (approximately 35 %) compared to TG (approximately 46 %).

**Conclusions:**

We conclude that segment-body vibrations applied in resistance training can offer an effective tool to increase maximum isometric force, compared to traditional training. The cause for these findings can be attributed to the additional local vibration stimulus.

## Key Points

- Maximum isometric force, range of motion, and muscle tension of hamstring muscle were compared between training regimes incorporating localized vibrations and traditional leg curl.
- The vibrational training group showed statistically significant improvements in maximum isometric force after the first week of training compared to 3 weeks for the traditional training regimen.
- The vibrational training group retained gain in performance for a longer time after the testing regimen than traditional training.
- The range of motion was improved, and muscle tension increase was less for the vibrational training group compared to the traditional training group.

## Background

Resistance training is considered as the bedrock for improving neuromuscular performance for both athletic and preventive health [1–7]. Over the recent past, establishing additional training protocols over a traditional resistance training routine has been a key focus of sports and conditioning experts [8–11]. In this paper, we specifically focus on enhancement through vibration training method which has recently emerged as an effective and convenient technique in improving neuromuscular performance especially when combined with a strength training program. Underscoring this trend, whole-body vibration, which is easy to administer through a vibrating platform, has gained tremendous popularity over the last decade for neuromuscular improvements [12–14]. For instance, it was found that when whole-body vibrations were induced through the feet using a vibrating platform into resistance training individuals, maximum isometric force of the corresponding muscle group improved between 6.5 % and approximately 40 % (for example, see [15, 16]). Two effects are believed to be the primary reasons for the increase of maximum force of vibration training compared to traditional training. First, Martin and Park [17] and Mester et al. [18] found that reflectory muscle contractions take place during the vibration stimulus. The sensitivity of muscle spindles increases during vibration training, and their effects on the stretch-shortening cycle are acute responses of the skeleton muscles to vibration training. It is assumed that consequently the reaction time and the threshold to create an action potential are decreased [19–21]. Park and Martin [22] identified an increase of muscle strength in hand flexor muscles while introducing segment-body vibrations to the tendons of the same muscles. Second, Eklund and Hagbarth [23] showed the antagonistic muscle relaxation during vibration, which improves motor coordination between agonist and antagonist [17]. As a consequence, the synchronization of neuronal activity between the muscles was improved, which resulted in increased maximum force [24, 25]. Rehn et al. [20] showed in their rating of 19 studies “ […] that there is strong to moderate evidence that long-term whole body vibrations can have positive effects regarding leg muscular performance” ([20], p. 4–5). It is furthermore reported that muscle tension (MT) increases after a traditional resistance training program [26]. No publication in English language could be identified to correlate muscle tension and vibration training.

In contrast to whole-body vibration, which only indirectly influences the training muscle groups, direct stimulation of a specific muscle group can be a good alternative candidate due to specificity and greater control. Only few studies examined the effect of vibration training called segment-body vibration on muscle strength and athletic performance [27]. These studies have reported an increase of the maximum force between 5 and 50 % (e.g., [24, 28, 29]). Therefore, the present study examined the effects of a special local vibration training, in which local segment-body vibrations were directly induced into the belly of the hamstring muscles in comparison to an identical training regime without vibrations. Both maximum isometric force and maximum flexibility as well as MT of the trained muscles were measured following a predefined period and regimen of training. Approved by the Ethics-Committee of the German Sport Univeristy Cologne.

## Methods

In the present intervention study, the leg flexor muscles were strengthened unilaterally. A total of 36 healthy subjects with at least 2 years’ experience in resistance training were selected for the study and randomized into a vibration training group (VG) and traditional training group (TTG). Note that no special prescriptions on diet and other behavioral activities were imposed for this study on any of the subjects. Additional informed consent was obtained from all patients for whom identifying information is included in this article. All procedures followed were in accordance with the ethical standards of the responsible committee on human experimentation (institutional and national) and with the Helsinki Declaration of 1975, as revised in 2008 [5].

The matched-pair technique [30] has been applied after the sixth pretest measurement to establish the two training groups. This technique assigned eight female subjects (44 %) to the VG and six female subjects (33 %) to the TTG. Both study groups performed the same exercise (leg curl) for the hamstrings on the right leg only. The setup of the leg curl machine in combination with the vibration generator physically only allowed superimposed vibrations during exercise of the right leg. The leg curl was performed on a lying leg curl machine (Gym 80, Signum Standart line, International Products, Gelsenkirchen, Germany [31]) with a standardized deadweight of 320 kg and a serially standardized weight stack of 85 kg. The weight stack did not limit the training load of any of the subjects. In addition to the regular resistance training machine, vibration was administered using a Medic Swing vibration generator (Mechatronic, Hamm, Germany). The device was convex and could be adjusted in height and angle, which allows its usage for local vibrations of lower body segments. Before participation, all subjects were fully informed of the purpose, experimental procedures, possible risks, and their rights and gave their written consent to participate.

### Variables and Statistics

The comparative performance metric for the current experiment was set to the following variables: isometric force, maximum range of motion (ROM), and maximum MT. The reliability of the measurements was measured beforehand in a test-retest design with 12 test persons during the pretests. The results of the test-retest scenario are as follows: isometric force *r* = 0.75, ROM *r* = 0.79, and MT *r* = 0.69 where *r* describes the correlation coefficient. Means and standard deviations were calculated by ANOVA with repeated measurements. Differences were tested with the Tukey post hoc test. Moreover, the different values between pre- and posttests were examined by dependent *t* test as well. The progression from pretest to retest (six measurements) within the two study groups was also recorded. Finally, the 1-Pearson correlation as a measure of similarity between the time course of the individual changes has been calculated at VG and TTG to present the means of maximum isometric force development from pretest to retest. Ward’s cluster algorithm has been applied at VG and TTG to present individual cases [32]. Sociometrical and anthropometrical data for this experiment are displayed in Table 1.

**Table 1:** Sociometrical and anthropometrical data of the subjects in study 1 ($\overline{\mathrm{x}}$ = mean, *s*_*x*_ = standard deviation)

	Females (*n* = 14)		Males (*n* = 22)	
	$\overline{\mathrm{x}}$	**s**_**x**_	$\overline{\mathrm{x}}$	**s**_**x**_
Age (years)	23.9	3.2	23.3	2.6
Height (cm)	165.6	8.9	188.7	8.2
Mass (kg)	63.0	3.7	83.5	6.9

### Testing

During the 2 weeks of pretesting phase, each subject additionally performed six measurements of all variables in order to exclude adaptations due to the familiarization of the testing protocol by the subjects. These test results are not part of the pretest. The actual pretest was performed prior to the first training with a rest interval of 72 h before the first session of training commenced. First, second, and third measurements and posttest have been performed after each training week with at least 24 h rest after the last training session. After 2 weeks of detraining, the same tests were repeated during the retest to measure changes in performance. Note that during this phase of testing, no vibrations were provided to the VG.

**Fig. 1:**
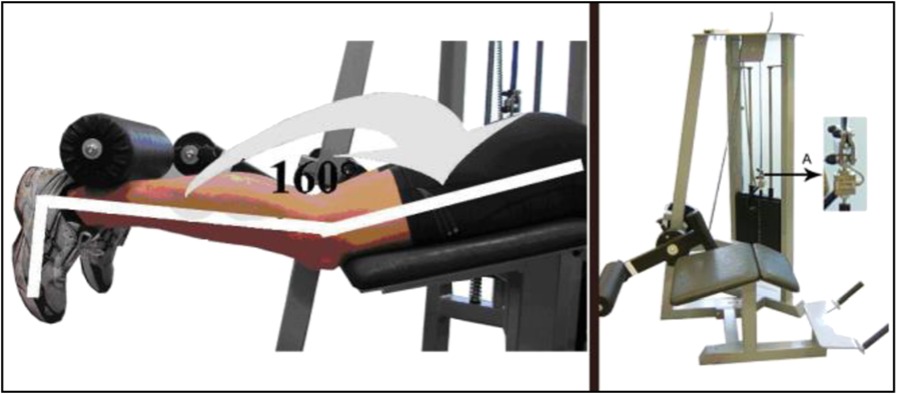
Measurement of maximum isometric force. *Left*: measurement position; *right*: leg curl machine with force sensor

The maximum isometric force of the leg flexors was measured using a leg curl machine (Gym 80 International, Gelsenkirchen, Germany), which was equipped with a force sensor (Mechatronic) (see Fig. 1, right part). The sample rate of the force sensor was 100 Hz, and the accuracy of measurement was 0.01 N. The time frame, during which the force was recorded, covered 5 s. Subjects took a facedown position during the measurement. The inner knee angle during measurements was 160° (see Fig. 1, left part).

**Fig. 2:**
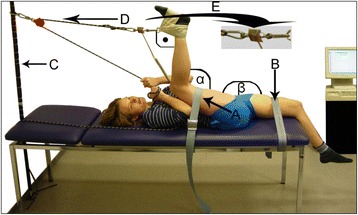
Measurement station and measurement procedure of muscle tension. *A*: fixation of the leg to be tested, *B*: fixation of the counter-lateral leg, *C*: adjustment pole, *D*: direction of traction, *E*: force sensor, target angle *α*, hip angle *β*, 90° between lower leg and cable winch

In order to determine the range of motion of the knee joint, the following novel setup has been applied considering the anatomical structures of the knee joint. To the best of our knowledge, no references record this setup. Subjects lay on their backs with the counter-lateral knee protruding over the edge of the bench. This leg was fixed with a belt (see Fig. 2b) to avoid evasive movement of the hip joint. The leg to be studied was brought to maximum flexion in the hip joint (hip angle β; see Fig. 2). The two-joint hamstring muscles (m. semitendinosus, m. semimembranosus, m. biceps femoris (caput longum)) were fixed in their final position at the hip joint (see Fig. 2a). The hip angle β was measured taking the horizontal plane and the distal end of the condylus lateralis as reference points and trochanter major as pivot point. The maximum hip angle β of the pretest is re-established during all following measurements and therefore standardized the measurement. While the hip joint angle is fixed, subjects used a cable winch to bring the leg to be studied into a stretching position. Consequently, the hamstring muscles experience an extension. The distal end of the condylus lateralis, the trochanter major of the hip joint, and the malleolus lateralis of the ankle form the reference points for the knee angle α (see Fig. 2). The measurement accuracy of the goniometer was 1°. Angles were recorded after 3 s in the maximum joint angle. The maximum knee angle was self-determined by the subjects and defined as a position which the volunteer felt discomfort, but no pain. The knee angle α of the pretest served as reference value for the muscle tension and therefore was established in all following measurements. After this standard value was achieved, subjects are asked to reach their maximum possible knee angle. This novel setup to measure the ROM of the knee joint to determine the extensibility of the hamstring muscles was applied to eliminate the pain that occurs while stretching the hamstring muscles with full knee extension [33–36].

While stretching the hamstrings, the muscle tension was measured using a force sensor (Mechatronic), which was located between the cable winch and foot loop. The force sensor and the lower leg of the subject were in a rectangular position. The force was recorded over a period of 5 s. The effect of gravity and the weight of the leg were eliminated from the maximum value measured in Newton. The following formula was used to calculate the actual muscle tension (the symbols explained in Table 2):

**Table 2:** Explanation of the parts of the formula used to convert the measured muscle tension into the actual muscle tension and their units

F1	Measured muscle tension in Newton (N)
*L*	Length of lower leg in centimeters (cm)
*f*	Weight of lower leg in Newton^a^ (N)
*α*	Knee angle in degrees (°)
*β*	Hip angle in degrees (°)
*δ*	Lever arm of force (60) = 0.035 m

^a^According to Leva (1996), with reference to studies by Zatsiorsky V and Seluyanov V [62], the percentage of the lower leg relative to the total body mass is 4.33 % in females and 4.81 % in males. The appraisal of the weight of the lower leg was based on these data

$$ \mathrm{Muscle}\ \mathrm{Tension}=\frac{F1*L+f*L* \cos \left(\alpha +\beta \right)}{\delta }. $$

### Training

**Fig. 3:**
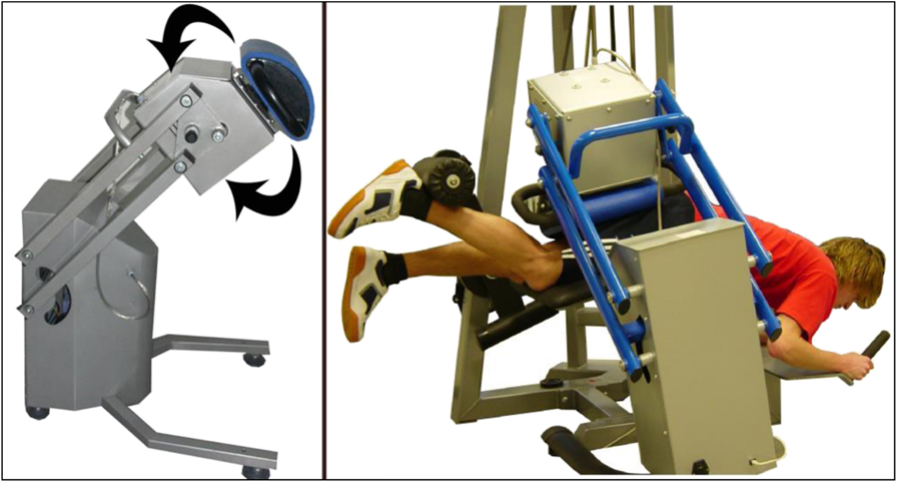
*Left*: vibration trainer Medic Swing (Mechatronic). *Right*: vibration training of the hamstrings

Both study groups performed the same exercise (leg curl) for the hamstrings on the right leg only. The subjects executed 4 training sets of 12 repetitions with 90 s pause between sets for all individuals of both groups. A total of three training sessions per week was carried out for a total of 4 weeks. The resistance progression was tracked using repeated measurements of the maximum isometric force as the test progressed. The VG group, in addition to the abovementioned protocol, also underwent vibrational loading on their hamstring. For the test persons in VG, the vibration area was placed directly on the center of the hamstrings (Fig. 3, right part). This device worked with a constant amplitude of 4 mm and allows a variable frequency between 18 and 38 Hz. Only the VG group trained with additional segment-body vibrations. Table 3 describes and summarizes in greater detail the training parameters for hamstring strengthening in both groups.

**Table 3:** Training parameters

Hamstring training	
Training load	4 series, 12 repetitions; with 70 % 1 RM for each arm
Pause	90 s between series
Training sessions per week	3
Training period	4 weeks
Hamstring training with additional vibration	
Frequency	18, 24, 30, and 36 Hz (1st, 2nd, 3rd, and 4th training week)
Amplitude	4 mm

The initial training load was determined at the end of the pretest phase, applying a test procedure previously described in detail [37]. The relative resistance was kept at 70 % of 1 RM for the whole training period in order to maintain a training load recommended for hypertrophy in resistance training [37, 38]. Consequently, the effective training load was adjusted according to the weekly measured maximum isometric force of the subject.

The resistance training followed a 5-min general warm-up procedure for both groups which included 3 min of treadmill running at 8 km/h, stretching of quadriceps and hamstring muscles (twice holding each stretch for 15 s alternating between each leg), and jumping exercises, which included skipping (6 m), two foot ankle hops (6 reps), and split squat jump (5 reps) [39].

## Results and Discussion

### Maximum Isometric Force

**Fig. 4:**
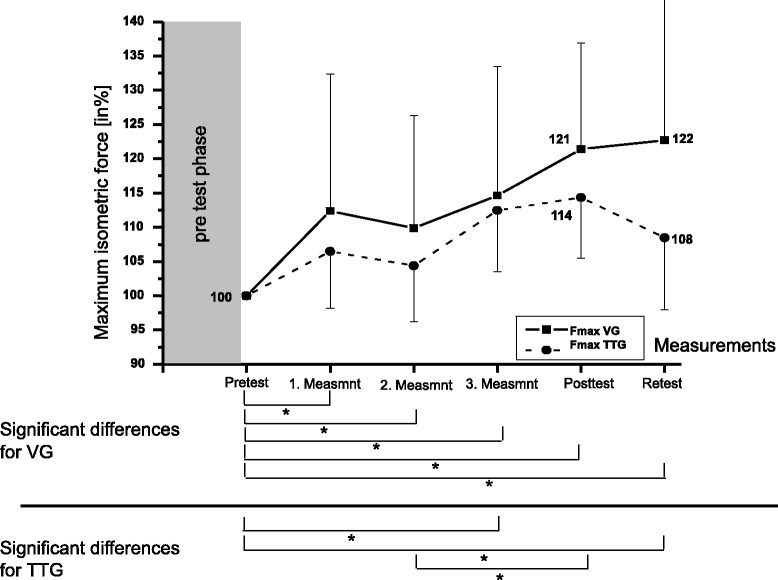
Development of maximum isometric force (Fmax) in VG and TTG (Tukey post hoc test). Significant differences (*α* = 5 %) between mean values of measurements were marked with an *asterisk*

The progression of the mean maximum isometric force (in percent) over the duration of the study is plotted in Fig. 4. The mean of the pretest values for both groups was normalized to 100 %. The force (*F*) values of 10.22 in the VG and 8.19 in the TTG became significant in the one-way ANOVA with repeated measurements. Thus, both interventions, i.e., strengthening with and without vibration, produced a significant increase on maximum force. However, more useful information could be gleaned using the post hoc test (Tukey) which provides information about the effects of the training time on maximum force.

Next, we studied at the *t* test for dependent samples for the changes in the groups from pretest to posttest (see Table 4). Both training groups showed significant changes between pretest and posttest. The mean maximum isometric force in the VG was 523 N in the pretest and 636 N in the posttest. For the TTG, the values were 567 N (pretest) and 648 N (posttest), respectively.

**Table 4:** Result of the *t* test for dependent samples for VG and TTG with increases of isometric maximum force

	MV	SD	Number	Diff	SoD	*t*	*df*	*p*	Fmax increase in %
VG pretest	523	193							21.4
VG posttest	635	196	18	−112	69	6.84	17	<0.05	
TTG pretest	567	194							14.2
TTG posttest	648	218	17	−81	57	4.89	16	<0.05	

*MV* mean value, *SD* standard deviation, *Diff* difference, *SoD* standard deviation of differences, *t t* value, *df* degrees of freedom, *p* probability, two-sided

### Results for Maximum Knee Angle

The variance analyses only revealed one significant *F* value (2.69) for the VG. The value calculated for the TTG (1.53) was not significant. Based on this result, we can assume that vibration-supported maximal strength training has an impact on the maximum knee angle. For the TTG, neither time of measurement nor training stimuli influenced the development of the maximum knee angle significantly.

The Tukey post hoc test for the VG showed that the development of maximum ROM shows only one significant difference. Thus, we can conclude that a vibration training of the hamstrings only has a limited impact on the maximum flexibility of the same musculature. The following *t* test for dependent samples provides more information about the development of the maximum knee angle over the duration of the training period (see Table 5).

**Table 5:** Result of the *t* test for dependent samples for the VG and TTG with increases in maximum knee angle (ROM)

	MV	SD	Number	Diff	SoD	*t*	*df*	*p*	Increase in max. ROM %
VG pretest	114	11							2
VG posttest	116	11	18	−2	4	2.38	17	<0.05	
TTG pretest	116	19							
TTG posttest	119	17	17	−3	6	2.01	16	n.s.	

### Maximum Muscle Tension

**Fig. 5:**
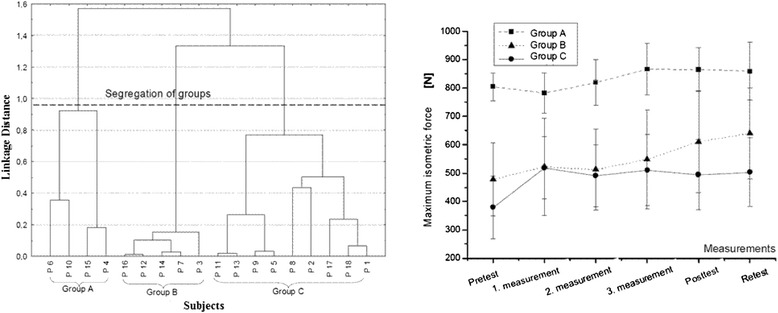
Development of maximum MT (muscle tension) of VG and TTG and results of the Tukey post hoc test. Significant differences (*α* = 5 %) between mean values of measurement are marked with an *asterisk*

Figure 5 illustrates the course of the mean muscle tension (MT) at maximum knee angle over the duration of the study. The conclusion we can draw from the significant *F* values for both groups (*F* = 8.1 for the VG; *F* = 6.91 for the TTG) is that maximal strength training with and without vibration influences the maximum ROM and maximum MT of the trained musculature. The Tukey post hoc test showed that the increase of MT in the posttest differed significantly from all previous measurements of VG. This is also true for the retest values of VG when compared to all other measurements except for the pretest. Similarly, we cannot assess any changes in MT for the TTG’s first 3 weeks of training (i.e., the pretest period). Statistically significant differences were measured between the pretest, first and third measurements, and posttest. These changes indicate an increase in MT at maximum knee angle. Finally, a significant decrease in MT can be ascertained between posttest and retest. The significant changes from the post hoc analysis were illustrated in Fig. 5. The subsequent *t* test for dependent samples showed the changes in both groups from pretest to posttest (see Table 6).

**Table 6:** Results of the *t* test for dependent samples for VG and TTG with increases in MT

	MV	SD	Number	Diff	SoD	*t*	*df*	*p*	Increase in MT in %
VG pretest	1205	606							34.3
VG posttest	1618	603	18	−413	394	4.45	17	<0.05	
TTG pretest	1156	681							46.0
TTG posttest	1688	864	17	−532	442	4.96	16	<0.05	

## Conclusions

These results, on maximum isometric force illustrated in Fig. 4, indicated significant variations over the whole training period. The results indicate that the maximum isometric force of the VG increased significantly from the pretest value even after the first training week. Thereafter, compared to the pretest, all the following measurements became significant. This can be contrasted with the TTG which achieved statistically relevant maximal force improvements only between the last three measurements and the pretest. Furthermore, the first significant difference in TTG is between the second measurement and posttest. This indicates that—in comparison to the VG—the improvement of maximum force occurred at a later time in the TTG. A possible reason for the faster response can be explained by an acute increase of training intensity through superimposed segment-body vibrations in VG [22]. The resulting higher load on the neuromuscular system in VG explained by the reflectory response forced by the vibration stimulus [18] may have caused a faster neuromuscular adaptation in VG compared to TTG.

**Fig. 6:**
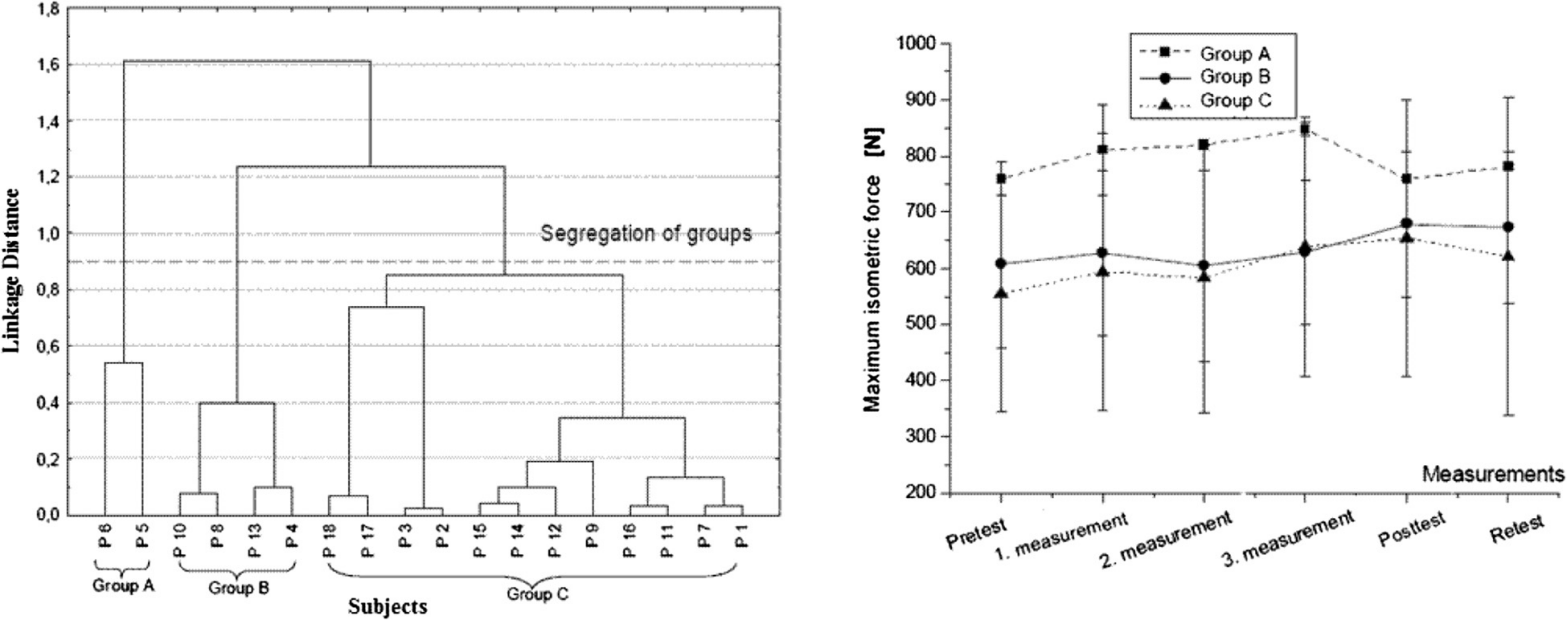
*Left*: tree diagram of Ward’s cluster analysis. Representation of groups of subjects (groups A, B, C) with similar maximum isometric force development from pre-retest of VG—group segregation after cluster analysis. *Abscissa* lists the subjects; *ordinate* shows the 1-Pearson correlation coefficient value (*r*) [31]. *Segregation line* was set at linkage distance 0.95. *Right*: presentation of mean maximum isometric force development of the three groups

**Fig. 7:**
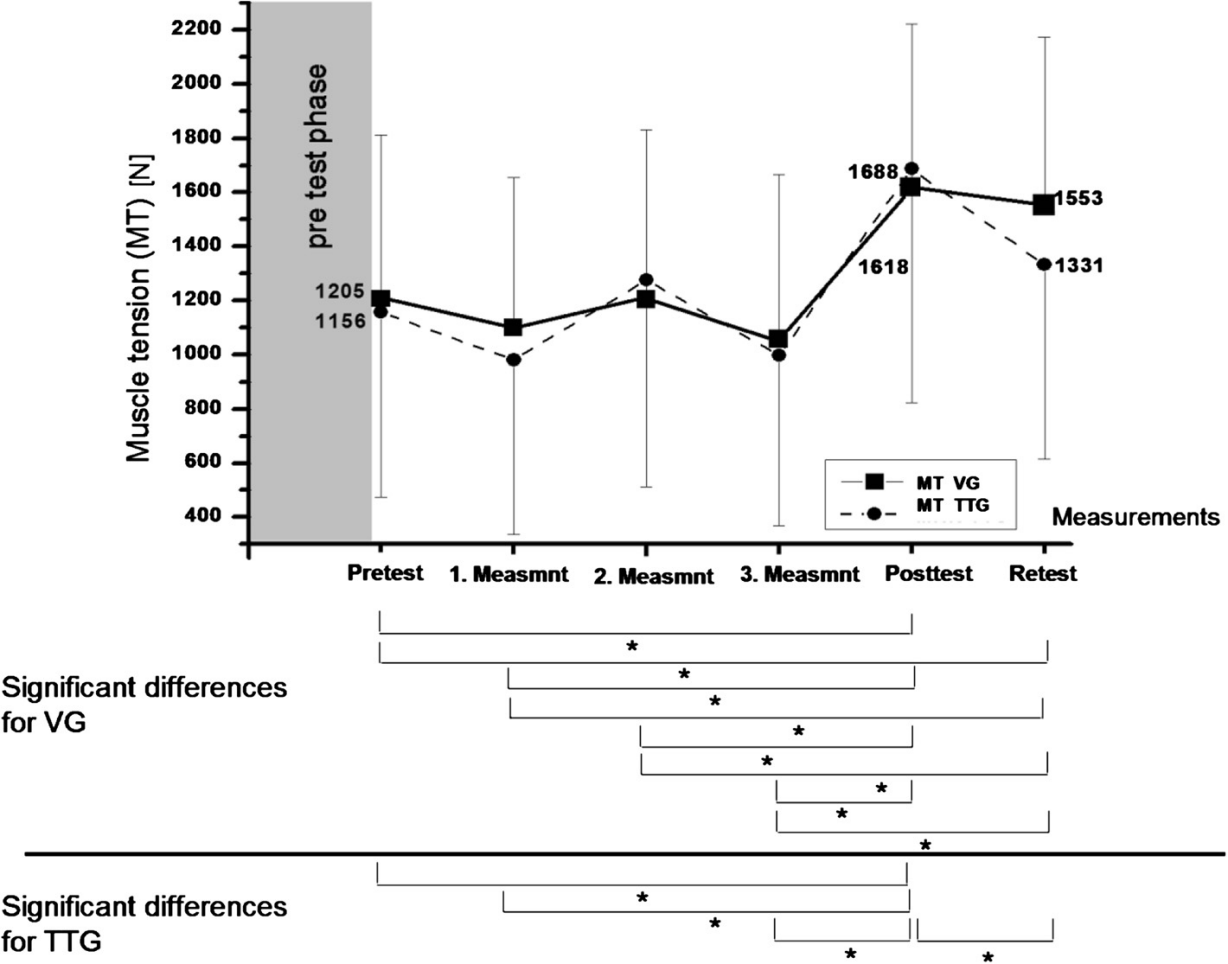
*Left*: tree diagram of Ward’s cluster analysis. Representation of groups of subjects (groups A, B, C) with similar maximum isometric force development from pre-retest of TTG—group segregation after cluster analysis. *Abscissa* lists the subjects; *ordinate* shows the 1-Pearson correlation coefficient value (*r*) [31]. *Segregation line* was set at linkage distance 0.9. *Right*: presentation of mean maximum isometric force development of the three groups

The *t* test (Table 4) shows that the mean increases in maximum force of 21.4 % (VG) and 14.2 % (TTG) were significant. A possible cause for this higher improvement rate of the VG likely stems from the additional vibration stimulus. In order to work out the practical relevance of this study, a cluster analysis was performed, and the results are summarized in Figs. 6 and 7. As a result, three groups (see Fig. 6, right) with distinct development levels became visible. The different courses of the mean values of all groups [1–3] are presented in Fig. 6 (right). For group A (four subjects), an initial decrease in maximum isometric force after the first week of training was observed. From the second measurement onwards, the maximum force value steadily increased compared to the posttest. In contrast to this development, group B (five subjects) showed a steady increase of the maximum force from pretest to retest. Finally, group C (nine subjects) experienced an initial steep increase in maximum force compared to the first measurement, which was subsequently followed by stagnation until the posttest.

The results for TTG subjects are plotted in Fig. 7 (left) with the dividing line at 0.9. Similar to VG subjects, TTG subjects were split up into three observational groups. The mean values for group A (two subjects) revealed an initial increase in maximum isometric force until the values dropped below the initial level in the post- and the retest. The slope of group B (four subjects) first showed a decrease in maximum isometric force to the second measurement. Afterwards, the maximum isometric force increased until it reached the peak at the posttest. Similar to group B of the VG, group C of the TTG (12 subjects) showed a continuously increasing maximum isometric force until the posttest. In summary, superimposed vibration during a submaximal resistance training on individuals with a comparably high strength level in hamstring muscles can initially decrease isometric force. A possible reason for this development is to be found in the higher absolute load of this training group, which might have caused an initial musculoskeletal overtraining to muscles, bones, and joints of the subjects [40]. In contrast, the same training method applied to individuals with a comparably average or lower strength level leads to an increase of isometric force after three training units. The effect of vibration training lasts also after 2 weeks of detraining for individuals of all entry levels. In comparison, a traditional submaximal strength training improves isometric force initially but reveals a potential loss of isometric force for comparably well-trained individuals after the third training week. Those results have to be treated with care, because the subjects have not been distributed equally in numbers to the groups A–C. The cluster analysis gives an overview about possible inter-individual different adaptations to a submaximal resistance training with and without superimposed segment-body vibrations. Further studies have to reveal the physiological reasons behind those differences.

Finally, the increase of maximum isometric force after 2 weeks of detraining at the retest remains significant at 22 % for the VG. Those results are in accordance with other findings [41–43], although little is known about the effects of segment vibration training on the magnitude and rate of strength loss during detraining. In contrast, the TTG significantly decreases the maximum isometric force during the same time period by 6 %. It is possible that the cause of the sustainability of the isometric maximum force level in VG can be found in the higher training intensity through the superimposed vibrations, and therefore a different regeneration time compared to the TTG of this population. It can be assumed that a change in isometric force manifested either earlier or later to the retest. Future studies should provide evidence for the effects of different detraining phases of vibration training. With regard to the maximum knee angle, from Table 5, a significant change from pretest to posttest can be noticed in the VG. Maximal strength training with induced vibration causes a higher increase in maximum ROM than traditional resistance training. However, the statistically relevant increase in the range of motion of 2 % plays a minor role in sports practice. Finally, for the maximum muscle tension, the data from Table 6 shows that the significant difference between pretest and posttest values becomes obvious in both groups. The mean increases in MT at maximum knee angle in VG (34.3 %) and TTG (46.0 %) were significant. This study reveals that a maximal strength training of the hamstrings brings about a significant increase of MT of this musculature.

These results thus indicate that dynamic strength training with additional local vibration stimulation represents an effective means to an increase of maximum isometric force of the hamstrings. In the VG, 21.4 % (112 N) of maximum isometric force was gained from pretest to posttest in contrast to 14.2 % (81 N) in the TTG. This vast increase of the VG is consistent with results reported in different sources (e.g., [44]; Knauf 1999, unpublished). Single cases with additional vibration stimulation show even higher increases between 13 % and 60 % of this parameter (e.g., [45, 16]). Clearly, the additional local vibration stimulus can be considered to be the reason for the much higher increase of maximum isometric force compared to the TTG. The fact that the vibrations are induced directly into the target muscle ensures that the generated amplitude and frequency reached the trained muscle without major transmission loss [46]. Moreover, since the frequency in the present study ranged between 18 and 36 Hz, a harmonic reflectory response of the involved muscles seems plausible [17] as well as a substantial relaxation of the antagonist [23]. Another explanation for the higher force development can be attributed to the reflectory contractions which lead to higher muscular activity [18]. In combination with submaximal loads (e.g., 70 % of the 1 RM), this causes an increase in force in the stimulated muscles [17] and a synchronization of muscular activity [24, 25].

Another important finding of this study was the long-term effects due to vibration training. In the retest, the maximum isometric force of the VG was still 22.7 % higher than at the time of the pretest (Römpke 2004 unpublished, [41]). In contrast to this, maximum isometric force of the TTG was 4 % lower in the retest than in the pretest. Since the training load was comparable in both groups, the increased intensity, which results from the additionally induced segment-body vibrations, obviously triggers a memory effect in the nerve-muscle system. The cluster analysis illustrated differences in the development of maximum isometric force between vibration training and traditional strengthening. Subjects of VG with high maximum force values in the pretest experience an initial decrease before they reach their late maximum in the retest. In contrast to this, the maximum force of the hamstrings of the subjects with high force values within TTG increased remarkably already after the first week of training. Another difference within TTG was the considerable decrease in the last week of training below the pretest value. Subjects with a low maximum isometric force showed an increase of this parameter within the first week of training (VG) and subsequently stagnated until the posttest, whereas the TTG revealed a continuous increase until the posttest. Obviously, the weaker athletes have difficulties coping with the vibration training stimulus. In the VG, the maximum knee angle increased significantly 2 % (2°) from pretest to posttest. The results of the TTG are almost identical (2.5 %, 3°). As a consequence, there were no better results produced using the additional vibration input. Among other factors, submaximal strength training generally improves recruitment, frequency (e.g., [47–49]), and synchronization of muscles and muscle groups. If the relaxation potential of the hamstrings can be thus enhanced, we can assume that the maximum range of motion increases. For the purpose of this study, the development of flexibility was of great importance. By means of repeated measurement, Mall et al. [50] were able to prove an increase in range of motion of 3.4°. Likewise, a study by Wydra et al. [51] showed an increase in passive range of motion of 8° within 2 weeks time. In this study, the tested leg performed a total number of 12 muscle stretches with a small improvement of 2°–2.5°. So the main reason for changes in maximum knee angle in this study can be found in the total number of 6 measurements during the whole study. MT at maximum knee angle also developed similarly in both training groups. MT remained almost unaltered from pretest until the third measurement, but increased afterwards. The increase increment of 34.3 % in the VG and 46.0 % in the TTG between pretest and posttest became significant in the *t* test. Since the maximum range of motion does not increase considerably between the third measurement and posttest, the development of MT must have a different cause. Submaximal strength training leads to both neuronal and structural adaptation processes in the musculature [52]. One of the results of muscle hypertrophy is an increase in parallel or serial sarcomere number, and thus an increase in the number of titin-myosin-complex filaments in the musculature which are part of every sarcomere [53]. The effects are supposedly related to an activation of more titin-myosin-complex filaments within one muscle (-group) which themselves increase the maximum MT [53]. As the maximum isometric force increases from the pretest to every measurement that follows and the MT increases after the third measurement, it is safe to assume that the process of muscle hypertrophy (consequently the changes in muscle structure) occurs following after the second training week. Since the time of the increase in MT is the same in both groups, it is also safe to assume that muscle hypertrophy occurs independently of the different training stimuli.

To the best of our knowledge, this is the first study to explore the effects of segment muscle vibrations superimposed to the muscle belly of the agonist muscle. The findings demonstrated that segment-body vibrations may improve muscle coordination, increasing the activation of the agonist muscle, resulting in an increase of maximum force. Therefore, we conclude that initially, the neuronal adaptations to submaximal strength training lead to an increase in maximum isometric force, but not to a significant increase in MT before the third measurement. This method thus offers to athletes a short-term increment of maximum force, which has the potential to improve overall performance in force-dependent sports. Resistance training in sports already covers a significant part of the training schedule. The application of segment muscle vibration in the present study increases the quality of the resistance training by achieving the same effects with segment muscle vibrations in shorter training time or achieving a higher isometric muscle force in the same training time. Moreover, evidence of positive effects of the application of whole-body vibrations in rehabilitations has been reported. Vibration training has been successfully applied in the rehabilitation process of anterior cross-ligament injuries [54–56], in treatment of knee ostearthritis [57], in rehabilitation of spina bifida [58], etc. In contrast, no evidence can be found that supports the use of vibration training in the rehabilitation process of stroke patients [59]. Those above rehabilitation applications are based on whole-body vibration. To the best of our knowledge, no studies about segment-body vibrations and rehabilitation exist. It is to be assumed that similar and more specific effects in rehabilitation can be achieved with segment-body vibrations. The role of muscle tension in static and dynamic force development is still under investigation. Force measurement under isometric conditions is influenced by muscle properties and neural factors [60]. The key limitation brought about due to lack of a control group can be alleviated if more studies are conducted, which include a control group and EMG. This can further reveal the role of muscle tension and would be the subject of future research. We will also like to point out another limitation of the study which stems from the fact that the bilateral effects of two different training methods of the two arms have not been controlled. It is known that unilateral resistance training of the elbow flexor muscles of the non-dominant arm of a test person can have a significant increase of isometric maximum force of 5.3 % at the contralateral elbow flexor muscles [61]. Thus, in summary, we conclude that segment-body vibrations applied in resistance training can offer an effective tool to increase maximum isometric force, compared to traditional training. Thus, as the training quality improves, higher benchmarks can be realized in the same period of training time compared to regular strength straining. The results of this study show the practical application of training method which is superior to traditional training by 7 % compared to traditional training.
