# Erratum to: Effect of Segment-Body Vibration on Strength Parameters

**DOI:** 10.1186/s40798-015-0028-6

**Published:** 2015-09-09

**Authors:** Ruben Tobias Goebel, Heinz Kleinöder, Zengyuan Yue, Ranajay Ghosh, Joachim Mester

**Affiliations:** 1 Sport Science Program, Qatar University, Doha, Qatar; 2 Institute of Training Science and Sport Informatics, German Sport University Cologne, Cologne, Germany; 3 The German Research Center, Center of Elite Sport, German Sport University Cologne, Cologne, Germany; 4 Department of Mechanical and Industrial Engineering, Northeastern University, Boston, MA USA

Unfortunately, the original version of this article [1] contained an error in the author list. The name of the author Ranajay Ghosh was incorrectly spelt as Ranajay Gosh. The correct spelling is Ranajay Ghosh.
